# Editorial: Innovations to improve screw fixation in traumatology and orthopedic surgery

**DOI:** 10.3389/fbioe.2022.1094813

**Published:** 2022-11-25

**Authors:** Jonas Widmer, Carl-Eric Aubin, G. Harry van Lenthe, Keitaro Matsukawa

**Affiliations:** ^1^ Department of Orthopedics, Balgrist University Hospital, Zürich, Switzerland; ^2^ Polytechnique Montréal, Montreal, QC, Canada; ^3^ Biomechanics Section, KU Leuven, Leuven, Belgium; ^4^ Murayama Medical Center (NHO), Musashimurayama, Japan

**Keywords:** spine fixaton, screw loosening, screw pull-out, bone screw, bone erosion, screw resilience

## Introduction

Although bony fixation with screws is a very common intervention and the technique has been refined in previous decades, insufficient screw hold and screw loosening still pose a relevant clinical problem with an incidence of about 10% in rigid fusion constructs. This rate is increased in motion-preserving instrumentations and in patients with low bone quality such as those with osteoporosis. In a recent study, the risk of screw loosening in vertebrae with low bone quality was found to be over 60% (Weiser et al., 2017). As a consequence, revision surgery is required in a substantial number of patients.

Improving screw fixation is a challenging field of research because a fundamental understanding of screw fixation in bone is still lacking. Conventional *in vitro* testing of the implant-bone structure using cadaveric bones is usually employed to evaluate the mechanical fixation of screws. Yet, the precise interplay between the screw thread and the intricate microstructure of trabecular bone is difficult to capture experimentally, especially right at the interface. Furthermore, experimental tests have demonstrated and quantified bone damage due to the screw insertion (Steiner et al., 2016), and microstructural finite element models have demonstrated that this can affect screw stability dramatically (Steiner et al., 2017). It is therefore an interplay of various factors that ultimately determine screw resilience in the bone.

Spine surgery is an area where screw fixation is particularly essential, but also particularly problematic. In the case of severe spinal deformity, a surgical instrumentation and fusion of the spine with implants anchored to the vertebrae and sometimes also to the pelvis is often performed. Pedicle screws have become the state-of-the-art fixation constructs for spinal fusion surgery (Lenke et al., 2008), with two complementary mechanical roles: 1) to apply forces to correct or reduce the spinal deformity intraoperatively and to maintain correction subsequently; 2) to create the proper mechanical environment for bony fusion. Spinal instrumentation constructs are subject to high loads under which pedicle screw fixation failure may occur (Abul-Kasim and Ohlin, 2014; Wang et al., 2016).

Spinal fusion is and always has been a race between biology and biomechanics. If fusion fails, eventually all spinal instrumentation either loosens or breaks. The spine cycles several million times a year, and the loads applied to the instrumentation and its fixation are highly variable (Spirig et al., 2021). They also depend on the construct design (anchor density (Widmer et al., 2020), anchor rigidity (Cornaz et al., 2021; Cornaz et al., 2022) use or absence of anterior column support (Burkhard et al., 2021), which could influence the outcomes and affect the risks of mechanical complications such as fixation loosening, material breakage, and adding on problems such as proximal junctional kyphosis or proximal junctional failure. Optimal construct stiffness is unknown. Too stiff constructs result in decreased load sharing and may limit fusion mass development and maturation. Too flexible instrumentation results in pseudarthrosis formation or early fatigue failure. Instrumentation design, sagittal balance correction, and choice of proximal fusion level have significant effects on the resulting forces in the spinal instrumentation and the success of osteosynthesis. Planning and optimization are important and can be aided opportunistically using analytical modeling (Widmer et al., 2020; Marie-Rosa et al., 2021) and biomechanical analysis (Wang et al., 2012; Wang et al., 2016; Bianco et al., 2017).

Using a combination of experimental and computational methods, this Research Topic will present novel insights and techniques that directly address the high complication rate of screw loosening in traumatology and orthopedic surgery:

## Spine fixations

Cornaz et al. quantified the contribution of the pedicle and corpus region in relation to bone quality and anchoring strength of pedicle screws. They demonstrated the importance of the pedicle region for screw hold, especially for reduced bone quality, and mentioned that selecting a larger screw diameter and augmenting the pedicle with bone cement may prevent screw loosening.

Similarly, Li et al. found that the regional bone property of screw holding plane mainly contribute to long-term screw fixation in anterior instrumentation in lateral lumbar interbody fusion technique. They emphasized the importance of optimizing screw trajectory and anti-osteoporosis therapy to reduce the risk of screw loosening.

In addition, Li et al. also illustrated the biomechanical effects of mismatch of the interbody support. According to their report, mismatch between the vertebral endplate and grafted bone caused mechanical stress around screws, suggesting the significance of modification of intervertebral cage design to maintain screw fixation, tailored to individual patient anatomy.

Meanwhile, Wang et al. reported a biomechanical study on interspinous process dynamic stabilization to reduce adjacent segment disorders, which is inevitable in spinal fusion surgery. This technology appears to alter kinematic motion less and has the potential to become an effective tool for spinal stabilization in the future.

## Fixations in non-spine related orthopedic areas

Bone screws are also used for prophylactic fixation in adult patients with an aggressive benign femoral neck lesion. Although the insertion of three cannulated screws is an established treatment method for nondisplaced femoral neck fractures in adults, it carries the risk of epiphyseal arterial vascular injury. Fu et al. investigated whether a technique using only two cannulated screws is biomechanically adequate to treat the femoral neck and does not result in screw avulsion. They show that with this technique adequate biomechanical strength can be achieved when the entire anterior cortical bone is involved.

Similarly, the stability of different screw trajectories for complex proximal humerus fractures was investigated by Mischler et al. in this research topic. Since the failure rate of locked plates is very high with the current state of the art, a new method with computationally improved screw trajectories was evaluated. Both finite element analyses and cyclic biomechanical testing showed a significant reduction in cut-out failure with the novel, proposed technique.

In calcaneus fractures, avulsion fractures of the tuber calcanei are characterized by a solid bone fragment at the Achilles tendon insertion. In an experimental study by Jordan et al. using synthetic bone, failure rates under cyclic loading were analyzed for different plate groups and screw-based fixation techniques. Surprisingly, the authors found that the 5.0-mm cannulated compression screws provided reliable stability and were a viable alternative to the commonly used 6.5-mm screws.

In the clavicle, hook plates are commonly used for dislocations of the acromioclavicular joint and fractures of the distal clavicle. Common complications resulting from this surgical technique with hook plates are subacromial bone erosion and peri-implant clavicular fractures. Wang et al. studied the effect of different clavicular hook plates, such as short plates, long plates, and posteriorly offset hook plates with different number and position of screws using finite element simulations. Based on their results, the authors discuss and show the trade-off between few screws and high loads at the clavicula and more screw but a higher risk of bone plate failure.

The authors who contributed to this Research Topic give a broad overview of the topic and the problem of screw fixations with a range of applications. Even though the application of screws is very diverse and covers a wide range of orthopedic specialty areas, the goal of achieving improved screw resilience is the same in all of them. Therefore, in the publication presented, we can also draw some overall conclusions from this Research Topic.

One major conclusion is that screws surrounded by higher bone density have higher resilience. This was shown in the study by Cornaz et al. who demonstrated increased retention in the pedicle at higher bone density for spine screws, in the study by Mischler et al. on the humerus who achieved better retention by adjusting the screw trajectory in areas of higher bone density, but also in the study by Fu et al. who indicated that anchoring of the screw in the cortex in the calcaneus is essential. Although this finding seems obvious, it appears to be of immense importance to incorporate it into current orthopedic techniques. Preoperative computer models that optimize and plan patient-specific screw trajectories, generic trajectories that lead to areas of higher bone quality, or implants that allow more targeted anchorage in cortical bone could therefore prevent problems with screws breaking out in the bone in the different areas.

Another conclusion is that the implant geometry of the screw going into the bone is very important. The implant geometry determines the resistance of the implant in the bone. This can be seen in our research topic, for example, in the study by Fu et al. in which different sizes and implants were tested and large differences in breakout force were found. Screws are primarily designed for an axial loading direction, but many of the load cases encountered in the application have a different primary loading direction, as shown by the many publications on our research topics from different fields. For example, Cornaz et al. and Li et al. point out that the loads acting on the screws during spinal fusion are mainly in the shear direction. New implant geometries such as nails and wedges that can be used as an alternative or in addition to screws could therefore be very promising to improve resilience. Much can therefore be achieved in the development of implants in the future.
